# The Effect and Feasibility of mHealth-Supported Surgical Site Infection Diagnosis by Community Health Workers After Cesarean Section in Rural Rwanda: Randomized Controlled Trial

**DOI:** 10.2196/35155

**Published:** 2022-06-08

**Authors:** Fredrick Kateera, Robert Riviello, Andrea Goodman, Theoneste Nkurunziza, Teena Cherian, Laban Bikorimana, Jonathan Nkurunziza, Evrard Nahimana, Caste Habiyakare, Georges Ntakiyiruta, Alexi Matousek, Erick Gaju, Magdalena Gruendl, Brittany Powell, Kristin Sonderman, Rachel Koch, Bethany Hedt-Gauthier

**Affiliations:** 1 Partners in Health Kigali Rwanda; 2 Center for Surgery and Public Health Brigham and Women's Hospital Boston, MA United States; 3 Program in Global Surgery and Social Change Harvard Medical School Boston, MA United States; 4 Department of Global Health and Social Medicine Harvard Medical School Boston, MA United States; 5 Epidemiology Department for Sport and Health Sciences Technical University of Munich Munich Germany; 6 Kirehe District Hospital Ministry of Health Kirehe Rwanda; 7 Ejo Heza Surgical Center Kigali Rwanda; 8 Rwanda Ministry of Health Kigali Rwanda; 9 Vanderbilt University Medical Center Nashville, TN United States

**Keywords:** obstetric surgery, community health workers, mobile health, surgical site infections, c-section, infection, community health, Rwanda

## Abstract

**Background:**

The development of a surgical site infection (SSI) after cesarean section (c-section) is a significant cause of morbidity and mortality in low- and middle-income countries, including Rwanda. Rwanda relies on a robust community health worker (CHW)–led, home-based paradigm for delivering follow-up care for women after childbirth. However, this program does not currently include postoperative care for women after c-section, such as SSI screenings.

**Objective:**

This trial assesses whether CHW’s use of a mobile health (mHealth)–facilitated checklist administered in person or via phone call improved rates of return to care among women who develop an SSI following c-section at a rural Rwandan district hospital. A secondary objective was to assess the feasibility of implementing the CHW-led mHealth intervention in this rural district.

**Methods:**

A total of 1025 women aged ≥18 years who underwent a c-section between November 2017 and September 2018 at Kirehe District Hospital were randomized into the three following postoperative care arms: (1) home visit intervention (n=335, 32.7%), (2) phone call intervention (n=334, 32.6%), and (3) standard of care (n=356, 34.7%). A CHW-led, mHealth-supported SSI diagnostic protocol was delivered in the two intervention arms, while patients in the standard of care arm were instructed to adhere to routine health center follow-up. We assessed intervention completion in each intervention arm and used logistic regression to assess the odds of returning to care.

**Results:**

The majority of women in Arm 1 (n=295, 88.1%) and Arm 2 (n=226, 67.7%) returned to care and were assessed for an SSI at their local health clinic. There were no significant differences in the rates of returning to clinic within 30 days (P=.21), with high rates found consistently across all three arms (Arm 1: 99.7%, Arm 2: 98.4%, and Arm 3: 99.7%, respectively).

**Conclusions:**

Home-based post–c-section follow-up is feasible in rural Africa when performed by mHealth-supported CHWs. In this study, we found no difference in return to care rates between the intervention arms and standard of care. However, given our previous study findings describing the significant patient-incurred financial burden posed by traveling to a health center, we believe this intervention has the potential to reduce this burden by limiting patient travel to the health center when an SSI is ruled out at home. Further studies are needed (1) to determine the acceptability of this intervention by CHWs and patients as a new standard of care after c-section and (2) to assess whether an app supplementing the mHealth screening checklist with image-based machine learning could improve CHW diagnostic accuracy.

**Trial Registration:**

ClinicalTrials.gov NCT03311399; https://clinicaltrials.gov/ct2/show/NCT03311399

## Introduction

Rates of cesarean section (c-section) births are increasing in low- and middle-income countries (LMIC), including in sub-Saharan Africa (SSA) [1]. Increased access to timely c-section can prevent maternal and neonatal mortality, but also carries risk of perioperative complications [2]. Surgical site infections (SSIs) are a significant cause of morbidity and mortality globally, but the magnitude of the risk is significantly higher in LMIC. In SSA, post–c-section SSI rates range from 7% to 48% [3-7], in part due to geographic and infrastructural barriers that delay or prevent patients from accessing care postoperatively [8,9].

In much of SSA, networks of community health workers (CHWs) provide home-based prenatal care to pregnant women, postpartum care for women after vaginal delivery only, and follow-up care for children under the age of 5 years [10]. However, women delivering via c-section can only access follow-up care at their local health center because CHWs are not currently trained to conduct home-based postoperative follow-up or wound care. In complementary work from our team, we found that geographical and financial barriers can lead to delays in return to care after discharge [8,11]. This delayed or lack of access to care may contribute to post–c-section SSI rates, which our group reported to be 10.9% in the district where this study took place [8]. Strengthening the CHW workforce to provide SSI screening and home-based care to women who deliver via c-section could reduce barriers to care and lead to earlier detection and treatment of SSIs. However, it is not known if home-based care by CHWs is feasible or improves access to care in this context. In LMIC, previous studies have demonstrated the feasibility of phone-based surveillance of postdischarge SSI, including in women who had undergone c-section surgery [12-15]. In our study, we explored the feasibility and impact of return to care of CHW-led SSI surveillance in patient homes using a mobile health (mHealth) checklist administered via REDCap (Research Electronic Data Capture; Vanderbilt University) either in person or via phone call to facilitate remote diagnoses. This mHealth screening protocol is a battery of questions about the presence or absence of clinical findings highly associated with SSI (eg, pain, swelling, discharge, and wound gaping), which we described in previous work [16].

In this paper, we describe two CHW-led mHealth interventions to diagnose SSIs following c-section in rural Rwanda, which are (1) administering a mobile phone–based SSI screening protocol to a patient via phone call; and (2) administering the same screening protocol, carried on an electronic tablet, in person during a home visit along with collecting wound photo images on the same tablet for remote diagnosis. Here, we compare these two interventions to the standard of care via a 3-arm randomized controlled trial (ClinicalTrials.gov NCT03311399) and describe the feasibility of the two interventions in this context.

## Methods

### Study Setting

This study was conducted in the Eastern Province of Rwanda at Kirehe District Hospital (KDH), a 233-bed facility operated by the Rwanda Ministry of Health and supported by Partners In Health, an international NGO. KDH serves a catchment area of 364,000 people including patients from Mahama Refugee Camp, which comprises over 50,000 people [17]. In Rwanda, over 91% of women deliver in health facilities [18]. Women in labor first present to their local health center where most vaginal deliveries take place. Complex cases and cases requiring surgical intervention are transferred to the district hospital to be assessed and managed by a general practitioner, who performs the c-section procedure, if indicated. After surgery, the woman is admitted to the postoperative ward for an average of 3 days for monitoring, medication administration, and wound checks. Before leaving the hospital, she receives postdischarge instructions directing her to the health center nearest to her home for follow-up and wound dressing changes.

### Study Population

This study included women aged ≥18 years who received a c-section at KDH between November 2, 2017, and September 4, 2018, and were residents of Kirehe District. Women who developed an SSI while being an inpatient or who remained inpatient at KDH past postoperative day (POD) 10 were excluded as they were not able to participate in a POD 10 home visit. Women who resided in Mahama Refugee Camp and were therefore not covered by the CHW network were also excluded.

### Preimplementation Procedures

We hired 4 study CHWs (sCHWs) using the Rwanda Ministry of Health criteria though a community-led process. The sCHWs received a 4-week training on implementation procedures, including the following: education on the Rwandan health sector; post–c-section follow-up; operating mHealth tools; best practices in wound photography; basic SSI physiopathology (including signs and symptoms of an infection); and how to examine surgical wounds and change wound dressings. Previously, we led a 7-month SSI protocol development study at KDH to identify a simple screening protocol with high accuracy to diagnose SSIs. In that study, three questions were found to have sufficient sensitivity and specificity for SSI diagnosis, which comprised the following: (1) fever since discharge from the hospital, (2) increasing pain since discharge, and (3) the presence of discolored wound discharge [19].

### Intervention Implementation

This randomized study included three arms—Arm 1, where the sCHW visited a participant’s home on POD 10 (SD 3 days) and administered the SSI screening protocol; Arm 2, where the sCHW called the participant and administered the SSI screening protocol over the phone on POD 10 (SD 3 days); and Arm 3, where the participant received the standard of care instructions to return to the health center for follow-up. In Arm 2, the sCHW attempted phone calls while sitting in the study office and made 3 call attempts before deeming a patient to be inaccessible. If a patient was deemed inaccessible, they were included in the evaluation of the feasibility of the intervention and classified as “not successfully assessed for SSI.” However, these individuals were not included in the evaluation of the presence of an SSI. If a participant in Arm 1 or 2 was suspected of having an SSI by the responses to the screening protocol, the sCHW was prompted to refer her to a nearby health center for additional medical care.

### Enrollment, Randomization, Follow-up, and Data Collection

All data collected were entered and stored using REDCap (v8.10.20), a secure web application certified for medical research studies [20]. The study staff enrolled and randomized eligible participants at discharge, independent of any patient factors, to one of the three study arms. The study staff prepared study packets in sealed envelopes numbered consecutively. REDCap was then used to randomly generate arm assignments to each packet using simple randomization in a 1:1:1 ratio [16]. All consenting participants’ demographic and socioeconomic data were collected using a self-reported questionnaire administered by a trained study data collector. In addition, the study staff extracted clinical data from the patients’ medical files. Upon discharge, the patients received a packet with arm-specific follow-up and general discharge instructions.

Each health center in the catchment area and KDH had a study-specific patient registry to document return to care. Study staff entered the following details into REDCap: return to care status, SSI diagnosis (by nurse), treatment received, hospitalization, patient referral, and need for surgical procedures, if any. Data collectors contacted each woman on POD 30 to validate what was captured in the registry and to ensure that no follow-up visits were missed.

### Statistical Analysis

Data were analyzed using Stata (14.0 version, Stata Corp) statistical software. We characterized study participant demographics and clinical characteristics using descriptive statistics. For the primary outcome, a patient was classified as having returned to care if the return visit was documented in the health center registry or if the patient reported returning to the health center during the POD 30 follow-up call. In this primary analysis, we excluded anyone without information on return to care by POD 30 from analyses. Feasibility assessments across Arms 1 and 2 were reported as the percentage of visits where that specific task was completed. We used a Fisher exact test at α=.05 significance level to assess the association between patients’ return to care and interventions implemented in Arms 1 and 2 as compared to Arm 3 (standard of care). We used a logistic regression model to assess the impact of study interventions on return to care, controlling for potential confounders that were unbalanced at baseline. In this primary analysis, we excluded anyone without information on return to care by POD 30 from analyses. We used chi-squared tests to assess for differences in having information about return to care by study arm and by patient demographics. We also conducted a sensitivity analysis, whereby any individual missing information on return to care was presumed to have not returned to care.

### Power

The estimated sample size was 364 patients per arm, for a total of 1092 patients. We anticipated an SSI rate of 15%, which would result in 55 SSIs per arm. Assuming an 80% return to care rate in the two intervention arms and a 40% return to care rate in the standard of care arm, we would have 81% power to detect a difference between the two intervention arms as compared to the routine care arm. The trial was halted when 1166 patients were enrolled (in excess of the targeted sample size of 1092).

### Ethical Considerations

Eligible women gave informed consent prior to participation. The study team members provided information in Kinyarwanda, including details of the three study arms and the right to withdraw from the study or refrain from giving information at any stage. Deidentified data were collected and managed using REDCap. This study was approved by the Rwanda National Ethics Committee (848/RNEC/2016) and Partners Human Research Committee (2016P001943/MGH). Seven months into the study, a Data and Safety Monitoring Board reviewed the study participants’ safety, data quality, and midterm outcomes, and deemed it appropriate to continue to study completion.

## Results

**Figure 1:**
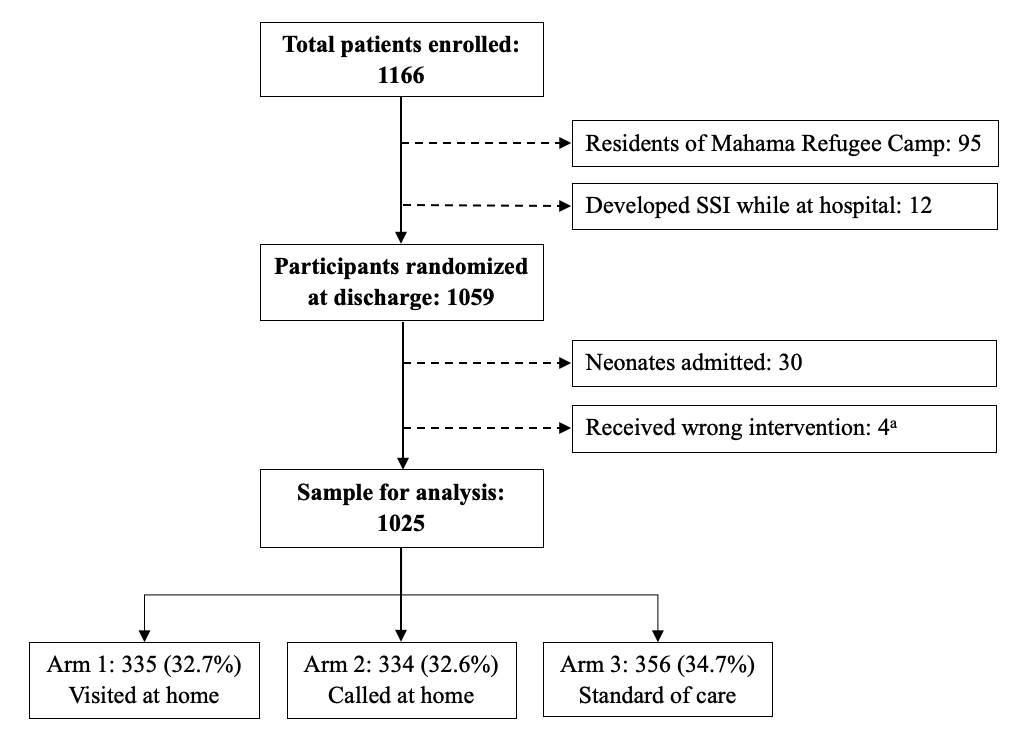
Flow chart of patient randomization into two treatment arms and one standard of care arm. a: one patient from Arm 1 received a phone call instead of an in-person visit, and 3 patients from Arm 3 received the in-person intervention despite being randomized into the control group; these 4 patients were excluded from analysis.

In total, 1166 women were enrolled, of which 107 (9.1%) were excluded—95 residents of Mahama Refugee Camp and 12 patients who developed an SSI while at the hospital. Of the enrolled patients who were randomized at discharge, 34 participants were removed from analysis—30 participants who remained in hospital after discharge to attend to their admitted neonates and 4 participants who were assigned to one arm but inadvertently received the follow-up of another arm. Of the remaining 1025 women, 335 (32.7%) were randomized to Arm 1, 334 (32.6%) to Arm 2, and 356 (34.7%) to Arm 3 (Figure 1).

There were no significant differences between the three groups for most demographic variables (Table 1), including age (*P*=.29), marital status (*P*=.2), occupation (*P*=.496), or type of insurance (*P*=.15). The only statically significant differences found were regarding education and income. Women in Arm 3 were more likely to report having only a primary education (*P*=.006). Women in Arm 2 were significantly more likely to report higher income (*P*=.03). There was no significant difference between the three groups in terms of their access to health care, measured by the cost of transportation from the woman’s home to the nearest health center (*P*=.93) and the travel time from a woman’s home to the nearest health center (*P*=.25; Table 1).

**Table 1:** Demographic characteristics of study participants by study arm (n=1025).

Characteristics		Total, n (%)	Arm 1: home visit (n=335), n (%)	Arm 2: phone call (n=334), n (%)	Arm 3: standard of care (n=356), n (%)	*P* value	
**Age (years)**							.28
	18-21	174 (17.0)	52 (15.5)	50 (15.0)	72 (20.2)		
	22-30	549 (53.6)	182 (54.3)	178 (53.3)	189 (53.1)		
	>30	302 (29.5)	101 (30.2)	106 (31.7)	95 (26.7)		
**Education**							.006
	No education	90 (8.8)	29 (8.7)	39 (11.7)	22 (6.2)		
	Primary education	696 (67.9)	225 (67.2)	205 (61.4)	266 (74.7)		
	Secondary education	214 (20.9)	75 (22.4)	82 (24.6)	57 (16.0)		
	University education	25 (2.4)	6 (1.8)	8 (2.4)	11 (3.1)		
**Marital status**							.2
	Single	95 (9.3)	28 (8.4)	26 (7.8)	41 (11.5)		
	Married	446 (43.5)	156 (46.6)	148 (44.3)	142 (39.9)		
	Living with a partner	480 (46.8)	150 (44.8)	160 (47.9)	170 (47.8)		
	Separated (divorced or widowed)	4 (0.4)	1 (0.3)	0 (0)	3 (0.8)		
**Occupation**							.496
	Student	10 (1.0)	2 (0.6)	5 (1.5)	3 (0.8)		
	Farmer	874 (85.3)	289 (86.3)	280 (83.8)	305 (85.7)		
	Employed	38 (3.7)	8 (2.4)	15 (4.5)	15 (4.2)		
	Self-employed	69 (6.7)	28 (8.4)	21 (6.3)	20 (5.6)		
	Housewife	34 (3.3)	8 (2.4)	13 (3.9)	13 (3.7)		
**Income^a^ (US $)**							.03
	>33.70	854 (83.3)	290 (86.6)	264 (79.0)	300 (84.3)		
	<33.70	171 (15.7)	45 (13.4)	70 (21.0)	56 (15.7)		
**Type of insurance**							.15
	No insurance	24 (2.3)	5 (1.5)	10 (3.0)	9 (2.5)		
	Community-based insurance	941 (91.8)	316 (94.3)	297 (88.9)	328 (92.1)		
	Private insurance	60 (5.9)	14 (4.2)	27 (8.1)	19 (5.3)		
**Cost of transportation from home to health center^a^ (US $; n=969)**							.93
	≤1.12	586 (60.5)	192 (60.0)	192 (61.3)	202 (60.1)		
	>1.12	383 (39.5)	128 (40.0)	121 (38.7)	134 (39.9)		
**Time from home to health center (n=964)**							.25
	≤1 hour	881 (91.4)	288 (90.0)	281 (90.7)	312 (93.4)		
	>1 hour	83 (8.6)	32 (10.0)	29 (9.4)	22 (6.7)		

^a^Calculated using an exchange rate of US $1 to 890 Rwandan Francs.

Of the 335 women in Arm 1, 295 (88.1%) were successfully visited in their homes and had the full SSI assessment completed by the sCHW (Table 2). The primary reasons for noncompletion in Arm 1 were prolonged hospitalization of either mother or baby or an inability to contact the mother to confirm the home visit appointment. Of the 334 women in Arm 2, 67.7% (n=226) were successfully called and assessed over the phone by a sCHW for an SSI. The primary reasons for noncompletion in Arm 2 were as follows: lack of mobile phone ownership, poor network coverage, or the phone belonging to another person (eg, husband or neighbor). Women in Arm 1 had slightly higher rates of reporting SSI symptoms as compared to women in Arm 2 (Table 3). As Arm 3 was the standard of care arm, there was no attempt to contact patients either via phone call or home visit.

**Table 2:** Feasibility of community health worker intervention arms.

Interventions and call attempts				Values, n (%)
**Home visit intervention (n=335)**				
	Number of patients who were visited and assessed for SSI^a^ by CHW^b^ at patient’s home		295 (88.1)	
	Home visits attempted by study CHW		295 (88.1)	
	**Completion of steps to conduct home visit**			
		Study CHW was able to find local CHW in patient’s village	281 (95.3)	
		Study CHW was able to locate patient’s home	295 (100)	
		Study CHW was allowed into patient’s home	295 (100)	
	Patient was at home when study CHW arrived		287 (97.3)	
	Patient allowed study CHW to ask SSI screening questions		295 (100)	
	Patient allowed study CHW to physically examine her		295 (100)	
**Phone call intervention (n=334)**				
	Phone call to patient attempted by study CHW		319 (95.5)	
	Number of patients who were called and assessed for SSI by CHW over phone		226 (67.7)	
**Phone call attempt #1**				
	Phone number went through, or phone rang (n=319)		268 (84)	
	Phone call resulted in talking with the patient (n=268)		167 (62.3)	
	**Outcomes of talking with patient (n=167)**			
		Patient answered SSI screening questions at time of call	163 (97.6)	
		Patient was busy	3 (1.8)	
		Patient did not respond, reason not recorded	1 (0.6)	
	**Reason for not talking with patient (n=101)**			
		Wrong number	7 (6.9)	
		Patient did not pick up the phone	6 (5.9)	
		Another person picked up the phone, patient was not available	87 (86.1)	
		Not reported	1 (1.0)	
	Patients requiring a second attempt (n=319)		156 (48.9)	
**Phone call attempt #2**				
	Number of patients who were called a second time (n=156)		133 (85.3)	
	Phone number went through, or phone rang (n=133)		89 (66.9)	
	Phone call resulted in talking with the patient (n=89)		51 (57.3)	
	**Outcomes of talking with patient (n=51)**			
		Patient answered SSI screening questions at time of call	50 (98)	
		Patient did not respond, reason not recorded	1 (2)	
	**Reason for not talking with patient (n=38)**			
		Patient did not pick up the phone	4 (11)	
		Another person picked up the phone, patient was not available	33 (89)	
		Not reported	1 (3)	
	Patients requiring a third attempt (n=156)		106 (67.9)	
**Phone call attempt #3**				
	Number of patients who were called a second time (n=106)		83 (78.3)	
	Phone number went through/phone rang (n=83)		36 (43)	
	Phone call resulted in talking with the patient (n=36)		13 (36)	
	**Outcomes of talking with patient (n=13)**			
		Patient answered SSI screening questions at time of call	13 (100)	
	**Reason for not talking with patient (n=23)**			
		Wrong number	1 (4)	
		Patient did not pick up the phone	2 (9)	
		Another person picked up the phone, patient was not available	20 (87)	

^a^SSI: surgical site infection.

^b^CHW: community health worker.

**Table 3:** CHW^a^ screening results by study arm (n=523)^b^.

Responses to CHW screening	Arm 1: home visit (n=295), n (%)	Arm 2: phone call (n=228), n (%)
Fever since discharge (n=522)	35^c^ (11.9)	22 (9.7)
Pain since discharge	51 (17.3)	32 (14.0)
Discolored drainage since discharge	46 (15.6)	20 (8.8)
CHW suspected wound infection	52 (17.6)	29 (12.7)
CHW advised patient to return to care (n=482)^d^	56^e^ (20.3)	30^f^ (14.6)

^a^CHW: community health worker.

^b^Arm 3 not included because no CHW screenings occurred in the standard of care arm.

^c^Missing data for 1 patient, n=294.

^d^Among those for whom CHW suspected wound infection in the home visit arm, 1 patient was not advised to return to care.

^e^Missing data for 19 patients, n=276.

^f^Missing data for 22 patients, n=206.

We had information on return to care for 896/1025 (87.4%) women, as described in Table 4. Women in Arm 2 were marginally, but nonsignificantly, more likely to have this information recorded (*P*=.06). There were no differences in having this documented among key demographics; though women with higher monthly incomes were more likely to have information on return to care recorded (*P*=.03). In the primary analyses, there was no difference in care-seeking behavior between the three arms. Women across all three arms had high rates of returning to clinic by POD 30 (99.7% in Arm 1, 98.4% in Arm 2, and 99.7% in Arm 3), with no significant statistical difference between them (*P*=.21 crude; *P*=.19 adjusted). Reasons for returning to care were not significantly different between the groups, with similar percentages of women returning for either routine wound care (n=253, 89.4% in Arm 1; n=264, 88.6% in Arm 2; and n=278, 90.3% in Arm 3; *P*=.08) or for a specific concern related to their c-section (n=30, 10.6% in Arm 1; n=34, 11.4% in Arm 2; and n=30, 9.7% in Arm 3; *P*=.08). There were similar rates of nurse-diagnosed SSIs in each group (n=33, 11.9% in Arm 1; n=34, 11.6% in Arm 2; and n=28, 9.3% in Arm 3; *P*=.54). In the sensitivity analysis, difference in return to care rates by study arm remained insignificant (*P*=.19 crude; *P*=.26 adjusted).

**Table 4:** Return to care behavior by 30-day post–c-section^a^ by study arm (n=896).

Outcomes			Total, n (%)	Arm 1: home visit, n (%)	Arm 2: phone call, n (%)	Arm 3: standard of care, n (%)	*P* value	
Patients randomized			1025 (100)	335 (32.7)	334 (32.6)	356 (34.7)	N/A^b^	
Patients^c^ with 30-day follow-up data			896 (87.4)	284 (84.8)	303 (90.7)	309 (68.8)	N/A	
**Source of 30-day follow-up data^d^**								
	Phone call with patient		555 (61.9)	180 (63.4)	188 (62.1)	187 (60.5)	.77	
	Health center registry		635 (70.9)	194^e^ (68.6)	215 (71.0)	226 (73.1)	.47	
	District hospital medical records		18 (2.0)	11 (3.9)	2 (0.7)	5 (1.6)	.02	
Patients^f^ who returned to care (n=896)			889 (99.2)	283 (99.7)	298 (98.4)	308 (99.7)	.21^g^	
**Among those with 30-day follow-up data (n=889)**								
	**Reason for returning to care**							.8
		Routine wound care (wound check and removal of stitches)	795 (89.4)	253 (89.4)	264 (88.6)	278 (90.3)		
		Concern related to c-section (fever, pain, and concern about wound)	94 (10.6)	30 (10.6)	34 (11.4)	30 (9.7)		
	Patient returned to care with nurse-diagnosed SSI^h^ (n=871)		95 (10.7)	33^i^ (11.9)	34^j^ (11.6)	28^k^ (9.3)	.54	

^a^c-section: cesarean section.

^b^N/A: not applicable.

^c^Those who were randomized.

^d^Information could have been collected from more than one source.

^e^Missing data for 1 patient (n=283).

^f^Those with 30-day follow-up data.

^g^*P*=.19 from likelihood ratio test (from logistic regression models controlling for education and income).

^h^SSI: surgical site infection.

^i^Missing data for 5 patients (n=278).

^j^Missing data for 6 patients (n=292).

^k^Missing data for 7 patients (n=301).

## Discussion

### Principal Findings

Surprisingly, nearly all patients in our study returned to care at least once by POD 30, with no significant difference in follow-up between arms. This contrasts with the findings in the Central African Republic, where a study reported that only 25% of surgical patients returned for a POD 30 follow-up visit [21]. A possible reason for this is that Rwanda, a small country with a strong functional decentralized public health system [22], offers greater access to follow-up care.

In this study, we observed that home-based follow-up care of participants allows the sCHW to enter the women’s homes, physically examine them, and take a photo of their wound. As close to 90% of participants in Arm 1 successfully visited and were assessed for SSI, we found that home visits are a feasible way to conduct post–c-section care. In rural Rwanda and many other low-resource settings, CHWs already provide in-home screening for child health [10], maternal health [23], and HIV care [24] referrals. The high feasibility of in-home screening could be due to the familiarity women have with the CHW system and how they value support from CHWs [24,25].

On the other hand, SSI screening by phone excluded close to 30% of women. Other studies in Tanzania [12] and Sudan [26] also demonstrated gaps in using telephone calls for postdischarge surveillance of SSIs. Currently, only 54% of households in Rwanda own a mobile phone [18]. Telephone-based interventions may be more feasible as phone access and network coverage expand. Two recent systematic review articles assessing the use of smartphones to identify SSI found that there are few articles in the literature, the majority are in high income settings, and they require smartphone ownership by patients. We have not found any other experience of CWH home follow-up for SSI identification and care [27,28]. Currently in the rural Rwandan setting, in-person sCHW visitation at the patient’s home provides greater follow-up coverage than phone calls alone.

Despite the null results in the difference between rates of return to care, this study has important implications linked to our understanding of the financial risks associated with health care seeking in this population. Undergoing a c-section is a financial burden for women in rural Rwanda [29]. This is true even for women covered by community-based health insurance. Our group has previously reported that the median out-of-pocket cost of transport for a single visit to the health center for women who received a c-section at KDH is up to 10% of their monthly income and that those who spent more money had increased risk of SSI [30]. Transportation cost was also self-reported by patients from Arm 3 of the study to be a barrier to health care seeking in the postoperative period [11]. These costs of transport were uniform across the arms of the study, as all three arms had equal rates of return to clinic.

In this setting, approximately 90% of patients do not develop an SSI. Treatment of an SSI is a principal reason for return to health center for care that cannot be provided by a CHW. Thus, accurate home rule-out of a post–c-section SSI can eliminate the need for 90% of women undergoing c-section to make the return journey and incur the out-of-pocket expense of transport to the health center. Given the financial burden of transport to the health center and the feasibility of in-person CHW SSI screening, leveraging the existing CHW system in Rwanda to bring postcesarean care to women’s homes could reduce both financial barriers to care and medical impoverishment. Further analysis is needed to determine the effect that home-based surgical wound monitoring would have on reducing unnecessary visits to the health center, health system cost savings, and workload on clinicians, though promising results have been shown in other settings [31,32]. This study’s findings will also be used to develop a supporting app to facilitate in-person SSI screening by CHWs.

### Limitations

This study had several limitations. Health center data may not have been consistent in quality due to variations in study patient tracking and data collection processes across sites. Targeted interventions, including calling health centers weekly with a list of expected enrolled patients and monthly in-person audits of each health center’s registry, were implemented to improve patient tracking. We also called all patients on POD 30 regarding their follow-up activities and SSI diagnoses. There was 100% agreement between data from registries and phone calls for the 228 patients from Arms 1 and 2 [19]. Secondly, we could not accurately or consistently capture dates of health center visits. As a result, we do not know when within-POD-30 women returned to care and whether there were differences between the intervention arms and the control arm. Additional research is needed to assess how CHW interventions affect the timeliness of return to care for post–c-section SSI evaluation.

### Conclusions

We did not observe a difference in the rate of return to the health center between women who were visited at home, who called at home, and who asked to continue with standard of care visits. In fact, women in all groups demonstrated high levels of health seeking behavior. However, our study found that home-based post–c-section follow-up by CHWs facilitated by an mHealth app to identify and refer SSIs is feasible. Our previous studies have shown that health center visits can pose a significant financial burden on women following c-section. Therefore, use of home visits for postoperative care could greatly reduce the nonmedical costs related to transport for routine follow-up for women who do not develop SSIs. Home, mHealth-enhanced, visits were also found to be more effective than phone-based follow-up for connecting CHWs with patients. Thus, home visits have the potential to greatly reduce the patient’s economic burden of post–c-section care. Future studies to understand the acceptability of CHW home visits for patients and health care workers are needed before this can be adopted as a standard care protocol.

## Appendix

Multimedia Appendix 1 CONSORT-EHEALTH (V 1.6.1) checklist.

## Abbreviations

c-section: cesarean section
CHW: community health worker
KDH: Kirehe District Hospital
LMIC: low- and middle-income countries
mHealth: mobile health
POD: postoperative day
RCT: randomized controlled trial
REDCap: Research Electronic Data Capture
sCHW: study community health worker
SSA: sub-Saharan Africa
SSI: surgical site infection
